# Complete genome sequence of *Methylobacterium radiotolerans* NYY1 isolated from Hong Kong soil

**DOI:** 10.1128/mra.00412-25

**Published:** 2025-08-25

**Authors:** G. K. K. Lai, N. Y. K. Yeung, K. M. Leung, F. C. C. Leung, S. D. J. Griffin

**Affiliations:** 1 Shuyuan Molecular Biology Laboratory, The Independent Schools Foundation Academy, Hong Kong SAR, China; Rochester Institute of Technology, Rochester, New York, USA

**Keywords:** *Methylobacterium*, bacteriochlorophyll, methanol dehydrogenase, photosynthetic bacteria

## Abstract

*Methylobacterium radiotolerans* NYY1, a Gram-negative, pink-pigmented facultative methylotroph, is a photosynthetic soil bacterium isolated in Hong Kong. Its complete genome, totaling 6,853,233 bp (G+C 71.05%) and including one circular chromosome, a 658 kbp chromid, and six plasmids, was established by hybrid assembly.

## ANNOUNCEMENT

Members of genus *Methylobacterium*, currently comprising over 50 named species (1), are Gram-negative, pink-pigmented (though sometimes yellow/orange), facultative methylotrophs (2). Typically plant-associated, they feature PQQ-dependent, Ca^2+^/La^3+^-binding Mxa/Xox methanol dehydrogenases (3) and often plant growth-promoting features, such as nitrogen fixation, phosphate solubilization, phytohormone production (4, 5), and antimicrobial activity (6). *Methylobacterium radiotolerans* (formerly *Pseudomonas radiora*), a species known for resistance to radiation and oxidative stress (7–9), is also photosynthetic, exhibiting bacteriochlorophyll, carotenoid production, and red-/blue-light photoreceptors (10–12). While *M. radiotolerans* shows great potential for crop protection (6) and bioremediation (13–15), its complete genome assemblies are rare because the species possesses a multipartite genome for adaptability (16, 17).

*M. radiotolerans* NYY1 was co-isolated with cyanobacteria from a sample of urban soil in Hong Kong (collected Oct. 2020, 22.264947N 114.129823E) using a spread plate method on BG-11 agar as described by Temraleeva et al. (18), incubating in an illuminated growth chamber (GC-300TLH, JeioTech, Korea) with a 12-h light cycle for 2 weeks. The purple colony was purified by single-colony streaking (>5 times) on nutrient agar. For DNA extraction, a single colony was streaked onto V8 agar (https://mediadive.dsmz.de/medium/J66) (on which NYY1 grows vigorously) and incubated for 48 h under similar illumination before harvesting around 200 µg cells from the plate for processing using a Qiagen DNeasy PowerSoil Pro kit following the manufacturer’s protocol. All incubations were at 25°C.

Paired-end short-read sequencing libraries were prepared using a Nextera XT DNA Library Preparation Kit (Illumina, Inc., USA) and sequenced via the Illumina MiSeq platform using v3 chemistry (2 × 300 bp). A total of 410,009 raw read pairs (SRX16018240) were quality filtered and trimmed using TrimGalore! v0.6.7 (stringency:3; -e:0.2), producing 406,255 read pairs (mean length 223 bp) totaling 90.6 Mbp (SRX24190812). Long-read libraries, prepared from the same extracted DNA using the Rapid Barcoding Kit SQK-RBK004 (without size selection), were sequenced on a Spot-ON Flow Cell (vR9), MinION sequencer, and MinKNOW v3.1.8 software, with base-calling by Guppy v2.1.3 high-accuracy mode (all by Oxford Nanopore). The 88,638 long-read data set (SRX16018241) was trimmed by Porechop v0.2.4 to give 51,550 high-quality reads (350 Mbp, mean length 6,790 bp, N50 8453) (SRX24190813) to scaffold the assembly. Default parameters were used for all software unless otherwise specified.

Hybrid assembly, overlap removal, and sequence rotation were performed by Unicycler v0.4.8-beta (19) yielding one chromosome, a chromid (20) and six plasmids (see Table 1). with a mean coverage of 51×, judged 98.55% complete (0.51% contamination) by CheckM v1.2.3 (21). The assembly was submitted to NCBI PGAP v5.3 (22) for annotation, using DFAST v.1.6.0 (23) for further analysis of individual replicons (see Table 1).

**TABLE 1:** The replicons of *Methylobacterium radiotolerans* NYY1 in comparison to *M. radiotolerans* JCM 2831 by average nucleotide identity (ANI)

Replicon	GenBank accession	Size /b.p.	Topology	% G+C	CDSs^*a*^	tRNA^*a*^	rRNA^*a*^ 5S-16S -23S	ANI *vs*. JCM 2831 replicons (CP001001-CP001009) as ANI% (alignment%) ^*b*^								
								Chromosome	Chromid pMRAD01	Plasmid pMRAD02	Plasmid pMRAD03	Plasmid pMRAD04	Plasmid pMRAD05	Plasmid pMRAD06	Plasmid pMRAD07	Plasmid pMRAD08
								6,077,833 bp	586,164 bp	47,003 bp	42,985 bp	37,743 bp	36,410 bp	27,836 bp	22,114 bp	21,022 bp
Chromo- some	CP090579	5,946,469	Circular	71.51	5617	69	4-4-4	98.73 (88.41)	70.66 (3.99)	0 (0)	70.33 (0.21)	75.87 (0.39)	67.37 (0.29)	70.50 (0.06)	0 (0)	0 (0)
Chromid pNYY1_1	CP090580	657,971	Circular	69.06	628	1	2-2-2	72.64 (18.48)	97.48 (69.24)	0 (0)	67.59 (0.16)	77.06 (0.51)	68.26 (0.47)	87.49 (0.22)	79.78 (0.03)	0 (0)
Plasmid pNYY1_2	CP090581	49,697	Circular	65.60	77	0	0	73.81 (9.24)	72.67 (2.65)	0 (0)	0 (0)	74.83 (0.88)	0 (0)	81.97 (0.36)	0 (0)	71.33 (0.87)
Plasmid pNYY1_3	CP090582	48,959	Circular	64.82	59	0	0	76.56 (18.08)	74.77 (4.04)	0 (0)	71.87 (2.47)	87.73 (5.74)	86.69 (3.84)	80.05 (0.87)	69.70 (1.76)	0 (0)
Plasmid pNYY1_4	CP090583	48,551	Circular	65.98	56	1	0	76.72 (13.67)	90.30 (4.37)	0 (0)	75.59 (9.19)	76.79 (3.87)	0 (0)	0 (0)	69.97 (2.06)	0 (0)
Plasmid pNYY1_5	CP090584	44,635	Circular	64.16	70	0	0	80.57 (6.98)	75.65 (4.33)	0 (0)	71.95 (1.45)	69.53 (2.67)	81.19 (0.98)	88.49 (2.29)	69.92 (0.52)	63.88 (1.91)
Plasmid pNYY1_6	CP090585	39,278	Circular	65.01	47	0	0	79.23 (11.88)	0 (0)	0 (0)	0 (0)	0 (0)	0 (0)	0 (0)	0 (0)	0 (0)
Plasmid pNYY1_7	CP090586	17,673	Circular	68.94	25	0	0	0 (0)	0 (0)	0 (0)	0 (0)	0 (0)	0 (0)	0 (0)	0 (0)	0 (0)

^*a*^ Annotation by DFAST v1.6.0 (16).

^*b*^ ANIs calculated by BLAST +v2.11.0 (24) in JSpecies v4.3.0 (25).

Gene clusters for bacteriochlorophyll synthesis, *bchFNBHLM* and *bchCXYZ*, are predicted at NCBI loci LZ599_09900 to LZ599_09875 and LZ599_14735 to LZ599_14720 respectively. Methanol dehydrogenase cluster *mxaFJGIRSACKLDEHB* (3, 26) is at LZ599_21700 to LZ599_21635, with proteins encoded at LZ599_02255 and LZ599_10320 similar to the La^3+^-dependent XoxF1 (27).

ANIb by BLAST+ v2.11.0 (24) in JSpecies v4.3.0 (25) found NYY1 and *Methylobacterium radiotolerans* strain JCM 2831 (GCA_000019725.1) to share an average nucleotide identity of 98.21%, although a comparison of replicons reveals some greater variation despite a similar genomic architecture.

## Data Availability

Sequence data for *Methylobacterium radiotolerans* NYY1 is available through NCBI under GenBank assembly GCA_021484845.1, with accession numbers for individual replicons given in Table 1.
